# Accuracy and precision assessment for activity quantification in individualized dosimetry of ^177^Lu-DOTATATE therapy

**DOI:** 10.1186/s40658-017-0174-7

**Published:** 2017-01-26

**Authors:** Gwennaëlle Marin, Bruno Vanderlinden, Ioannis Karfis, Thomas Guiot, Zena Wimana, Patrick Flamen, Stefaan Vandenberghe

**Affiliations:** 1 Department of Medical Physics, Institut Jules Bordet-Université Libre de Bruxelles (ULB), 121 Boulevard de Waterloo, 1000 Brussels, Belgium; 2 Department of Nuclear Medicine, Institut Jules Bordet-Université Libre de Bruxelles (ULB), 121 Boulevard de Waterloo, 1000 Brussels, Belgium; 3 Medical Imaging and Signal Processing (MEDISIP), Department of Electronics and Information Systems (ELIS), Faculty of Engineering and Architecture (FEA), Ghent University (UGent), 185 De Pintelaan, 9000 Gent, Belgium

**Keywords:** Precision, Accuracy, ^177^Lu, SPECT/CT, Well counter, Radionuclide calibrator

## Abstract

**Background:**

In order to obtain a reliable ^177^Lu-DOTATATE therapy dosimetry, it is crucial to acquire accurate and precise activity measurements with the radionuclide calibrator, the SPECT/CT camera, and the NaI(Tl) well counter. The aim of this study was to determine, in a clinical context, the accuracy and the precision of their activity quantification over a range of activities and time.

Ninety-three ^177^Lu sources from the manufacturer were measured in the radionuclide calibrator over 2.5 years to evaluate its calibration accuracy and precision compared to the manufacturer’s value. A NEMA 2012/IEC 2008 phantom was filled with a ^177^Lu activity concentration sphere-to-background ratio of five. It was acquired with the SPECT/CT camera to determine the reconstruction parameters offering the best compromise between partial volume effect and signal-to-noise ratio. The calibration factor was computed accordingly. The calibration quality was monitored over 2.5 years with 33 phantom acquisitions with activities ranging from 7040 to 0.6 MBq. Home-made sources were used to calibrate the well counter. Its reliability was evaluated with activities ranging from 150 to 0.2 kBq measured 34 times over 2.5 years.

**Results:**

For the radionuclide calibrator, median [interquartile range] for the error on activity measurement was −0.99 [1.31] %. The optimal SPECT reconstruction parameters were obtained with 16 iterations, 16 subsets and a 12-mm Gaussian post-filter. The calibration factor was 9.87 cps/MBq with an error of −1.05 [2.12] %. The well counter was calibrated with 31.5 cps/kBq, and the error was evaluated to −12.89 [16.55] %.

**Conclusions:**

The accuracy and the precision of activity quantification using dedicated quality control were found to be sufficient for use in dosimetry implemented in clinical routine. The proposed methodology could be implemented in other centres to obtain reproducible ^177^Lu-based treatment dosimetry.

## Background

Overexpression of somatostatin receptors, a hallmark of well differentiated metastatic neuroendocrine tumors (NETs) [1], renders NETs susceptible to treatment with radiolabeled synthetic somatostatin analogues. According to recently established guidelines, peptide receptor radionuclide therapy (PRRT) consists in the systemic administration of fixed activities of radiolabeled somatostatine analogues, usually fractionated in sequential cycles of 6–12 weeks [2]. Third generation PRRT consists of DOTA(0), Tyr(3)-octreotate labelled with radionuclide ^177^Lu (^177^Lu-DOTATATE) and has proven efficacy in clinical trials [3] with a low toxicity profile.

^177^Lu represents an interesting therapeutic radionuclide with a physical half-life of 6.647 days and a combined electron and photon emission. ^177^Lu has a 100% probability of decaying via electron emission to three excited levels of ^177^Hf. The most abundant electrons have a maximum energy of 177, 385 and 498 keV and an emission probability of 11.58, 9.1, and 79.3%, respectively [4, 5]. The high energy transfer and the short track of these electrons enable ^177^Lu to be used in molecular radiotherapy of metastatic cancer. In addition, the transitions from the three excited levels of ^177^Hf to its ground state occur via six gamma photon emissions. The two most abundant photons have energies of 112.95 and 208.37 keV with 6.17 and 10.36% probability of emission, respectively [4, 5]. These photons can be used for imaging on a SPECT camera which offers unique dosimetry possibilities.

Understanding the correlation between the tumor cumulative absorbed dose and tumor response is crucial for the optimization of PRRT outcome. The absorbed dose to the tumor is restricted by the maximum acceptable absorbed dose to the kidneys and bone marrow (i.e., the therapy critical organs), hence limiting the total number of administered cycles. A reliable dosimetry method is thus mandatory in order to improve the efficacy/toxicity balance and achieve patient-specific treatment planning. More specifically, pre- and peri-therapeutic dosimetry might help us gain better knowledge of PRRT therapeutic window, hence, allowing the prediction of the maximal absorbed dose to the tumors while minimizing radiation burden to the critical organs.

In order to achieve a reliable dosimetry, accurate and precise activity quantification is required. Quantitative data suitable as inputs for dosimetry are obtained through different measuring devices: the radionuclide calibrator to measure the administered activity, the SPECT/CT camera to acquire images of the radiopharmaceutical distribution, and the NaI(Tl) well counter to quantify activity in blood samples over time. In the last decade, optimization of acquisition and reconstruction parameters for ^177^Lu quantitative SPECT imaging have been extensively studied in order to improve the absorbed dose computation reliability [6–10]. The other aforementioned measuring devices, though also involved in PRRT dosimetry, were often overlooked.

The aim of this work was to determine, in a clinical context, the accuracy and the precision of all activity quantification methods in order to develop a reliable ^177^Lu-DOTATATE dosimetry. The first objective was to determine the most adequate parameters for ^177^Lu activity measurement with the radionuclide calibrator, the well counter and the SPECT/CT camera used in clinical routine. The second objective was to determine a calibration factor (CF) to convert counts to becquerels for each device. Finally, dedicated quality control steps were developed to evaluate the accuracy and the precision of the established CFs for the chosen measurement parameters with respect to manufacturer maintenance, time, and range of activities.

## Methods

For the radionuclide calibrator and the SPECT/CT camera, the standard quality controls were performed according to national guidelines (i.e., Federal Agency for Nuclear Control recommendations, already available from 2008 and transposed into Belgian law on March 15, 2016).

Based on the given manufacturer’s specifications (ITG, Isotope Technologies Garching GmbH), non-carrier added ^177^Lu had an activity measurement error maximum of 5%, a radionuclide purity above 99.9% with a ^177m^Lu/^177^Lu ratio lower than 10^−7^.

### Radionuclide calibrator

#### Description

All prepared activities were measured with a radionuclide calibrator CRC-15R (Capintec; manufactured in 1990) with an ionization chamber well of 6 cm diameter and 26 cm depth. ^177^Lu vials were put at the bottom of the dedicated syringe holder raised at 3 cm relative to its normal position in order to place the vials closer to the area of highest sensitivity.

#### Calibration with ^177^Lu

The radionuclide calibrator was calibrated for ^177^Lu with a vial from ITG with a total amount of activity of 15.3 GBq as measured by the manufacturer. The CF was then adjusted to reach the expected activity decay-corrected at the time of measurement.

#### Quality control

A specific quality control related to ^177^Lu was implemented to evaluate the validity and control the constancy of the CF: the activity measured by the manufacturer was compared to the on-site measurement for each received ^177^Lu ITG vial.

### SPECT/CT camera

#### Description

**Fig. 1:**
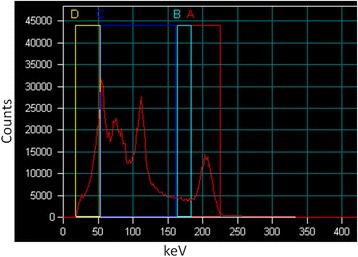
^177^Lu energy spectrum acquired on SPECT/CT camera. Four energy windows were defined but only the 208 keV photopeak window A (20% width [187.56–229.24 keV]) and the lower scatter window B (10% width [166.72–187.56 keV]) were used

All acquisitions were performed on a hybrid dual head SPECT/CT camera (Symbia TruePoint T, Siemens Healthcare; manufactured in 2008) with a 9.5 mm NaI(Tl) crystal thickness and a rectangular field of view of 537 × 383 mm^2^. The acquisitions were all performed with the parallel-hole medium energy, low penetration (MELP) collimator in non-circular (auto-contour) step-and-shoot mode. 32 frames (180°) of 40 s were acquired by each head; the time per frame was doubled for very low count rates (i.e., lower than 20 kBq/mL for the phantoms and for the last SPECT/CT acquisition at 168 h post-injection for the patients). One photopeak energy window centred on 208 keV and one lower scatter window were used, their widths were 20% [187.56–229.24 keV] and 10% [166.72–187.56 keV] of the energy peak, respectively (Fig. 1). The SPECT acquisition was followed by a CT scan. The parameters of the tube were a voltage of 130 kV and an effective current-time product of 40 mAs (CareDose, Siemens Healthcare). CT data were reconstructed to 512 × 512 matrix with 0.98 × 0.98 mm^2^ pixel size and a slice thickness of 5 mm. Out of the three CT reconstruction filters available on the Symbia Truepoint T and dedicated to body (B) image acquired with standard (s) scan mode (z-flying focal spot technology without hultrahigh resolution comb filter) (B08s, B30s and B60s), two were selected, namely, B08s and B30s. The very large smoothing filter B08s was used for attenuation correction (AC), and the B30s was used for volume of interest (VOI) delineation in soft tissue.

Reconstruction of the data was carried out with the software from the camera manufacturer (Syngo MI Applications version 8.5, Siemens Healthcare). A SPECT matrix size of 128 × 128 was used with a voxel size of 4.8 × 4.8 × 4.8 mm^3^. For SPECT data, the proprietary iterative (ordered subset expectation maximization or OSEM) reconstruction algorithm with collimator depth-dependent three-dimensional resolution recovery (Flash 3D, Siemens Healthcare) was chosen. No pre-filtering of the images was applied. CT based AC and dual energy window (DEW) scatter correction (SC) were used but no additional partial volume effect correction was performed. The SC was performed as follows:

$$ {C}_{\mathsf{sc}\ \mathsf{corrected}}={C}_{\mathrm{Ph}}- k\times {C}_{\mathrm{LW}} $$

with ${C}_{\mathsf{sc}\ \mathsf{corrected}}$ the number of counts after SC, *C*_Ph_ the number of counts in the photopeak window, *C*_LW_ the number of counts in the lower scatter window and *k* a scaling factor (equal to 1 in this study).

**Fig. 2:**
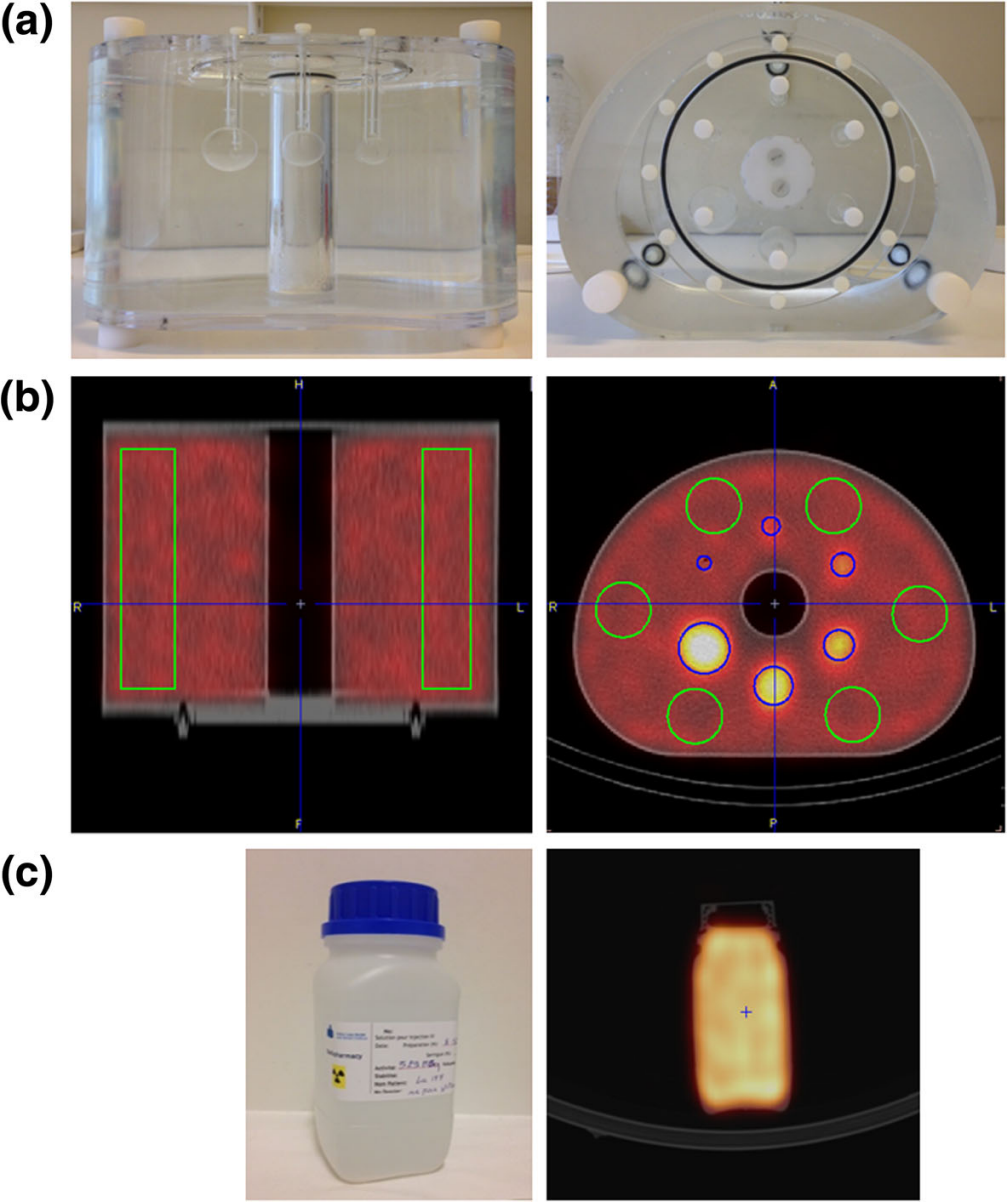
**a** NEMA 2012/IEC 2008 phantom. **b** Six cylindrical VOIs (*green*) were defined in the main compartment of the NEMA phantom on SPECT/CT images for the SPECT CF computation; for the reconstruction parameter determination, six spherical VOIs (*blue*) were added. **c** Photograph and SPECT/CT image of the plastic bottle used for the CF quality control

The determination of the number of iterations, the number of subsets, and the size of the Gaussian post-filter for OSEM reconstruction was based on a phantom study. The NEMA 2012/IEC 2008 PET body phantom was used (Fig. 2). The main compartment and the spheres (10, 13, 17, 22, 28, and 37 mm diameter) were filled with 193 and 1012 kBq/mL of ^177^Lu, respectively, to mimic the typical contrast between the liver and the hepatic lesions in patients treated with ^177^Lu-DOTATATE. The central cylinder was filled with air. The phantom was acquired with the previously defined parameters. The images were then reconstructed with a Gaussian post-filter with a full width at half maximum (FWHM) of 0, 4.8, 9.6, 12, and 14.4 mm and with a product of iterations (i) and subsets (s) equal to 4i × 8s, 8i × 8s, 8i × 16s, and 16i × 16s. With PMOD software (PMOD biomedical image quantification version 3.6), six cylindrical VOIs (212 mL each) were drawn in the main compartment, and six spheres were defined according to their theoretical dimensions and positions (Fig. 2). The noise in the homogeneous area was evaluated with the mean coefficient of variation (CoV) measured in the cylinders. The partial volume effect on activity quantification in small areas was assessed by means of the activity concentration ratio between the 22 and the 37 mm spheres. Taking into account that a large number of iterations and subsets with a small filter decreases the partial volume effect but increases the noise, several sets of parameters offered a good compromise between both resolution and noise. The optimal parameters were chosen by way of visual assessment of the reconstructed phantom images by a blinded expert nuclear medicine physician.

This phantom and the others presented in this study were all prepared with addition of an excess of ethylenediaminetetraacetic acid (EDTA) to prevent ^177^Lu from sticking to the phantom walls. The syringes used to fill the phantoms were systematically measured in the radionuclide calibrator before ^177^Lu injection in the phantom and afterwards to compute the exact amount of injected activity. The phantoms were shaken before each acquisition to prevent ^177^Lu from sedimenting. All the measurement and acquisition dates and times were carefully reported.

#### Calibration with ^177^Lu

For the determination of the CF for SPECT images with ^177^Lu, the NEMA 2012/IEC 2008 PET body phantom was used (Fig. 2). The main compartment, the central cylinder, and the spheres were filled with 686, 1010, and 1668 kBq/mL, respectively. The approximate total amount of ^177^Lu activity in the phantom was 7040 MBq, which is close to the activity injected to the patients for therapy. The phantom was acquired at ten time-points during 1.5 months. Six cylindrical VOIs (212 mL) were distributed in the main compartment on each SPECT/CT acquisition (Fig. 2).

For each VOI, the CF expressed in (cps/voxel)/(MBq/ml) was then computed as follows:

$$ \mathrm{C}\mathrm{F}=\frac{c_{\mathrm{mes}}}{T_{\mathrm{acq}}\times {C}_{\mathrm{prep}}\times exp\left(\frac{- \ln 2\times \varDelta t}{T_{1/2}}\right)\ } $$

With *c*_mes_ the mean number of counts per voxel, *T*_acq_ the acquisition duration (i.e., time per frame multiplied by total number of frames), *C*_prep_ the prepared activity concentration, *Δt* the time between the phantom preparation and the acquisition, and *T*_1/2_ the physical half-life of the radionuclide. To be more easily compared with other studies, the voxel size was taken into account: after multiplying the previous CF by the number of voxels per mL, the CF was expressed in cps/MBq. The final CF was computed by averaging the 60 computed CFs (6 cylindrical VOIs × 10 acquisitions).

#### Quality control

Some specific quality controls related to the SPECT CF were developed to evaluate its stability over time and range of activities and determine the influence of manufacturer maintenance. For this purpose, a phantom was filled with ^177^Lu every 9 months (Table 1). This procedure was simplified by using a less complex phantom consisting of a 6.5 × 6.5 × 15 cm^3^ plastic bottle (Fig. 2). The exact volume in the bottle was obtained by weighing before and after filling with water. The NEMA phantom was used for the last verification in March 2016. The phantoms were acquired multiple times on the SPECT/CT camera during more than 1 month to cover a large range of activities. The first phantom was acquired twice on the same day, before and after a complete tuning of the photomultipliers to evaluate the impact on the quantification. For each acquisition, a large box was drawn with PMOD around the bottle or in the main compartment of the NEMA phantom. The measured activity was computed with the determined CF. The error was evaluated by comparing this result to the expected activity based on the radionuclide calibrator measurement corrected for physical decay.

**Table 1:** Characteristics of ^177^Lu phantoms acquired on SPECT/CT camera to check for CF stability over time and range of activities

Phantom type	Bottle			NEMA
Phantom preparation date	Mar 2014	Dec 2014	Oct 2015	Mar 2016
Activity at first acquisition [MBq]	600	530	330	1926
Activity at last acquisition [MBq]	17	2	0.6	37
Activity concentration at first acquisition [kBq/ml]	933	815	526	174
Activity concentration at last acquisition [kBq/ml]	27	3	1	3
Delay between first and last acquisition [days]	34	55	60	38

### NaI(Tl) well counter

#### Description

The measurement of the activity in samples was performed with a well counter 2480 WIZARD^2^ (Perkin Elmer; manufactured in 2011) with a single 7.5 cm diameter NaI(Tl) crystal well and a solid lead shield of 7.5 cm. Standard tubes (7.5 cm height × 1.2 cm diameter; 8 mL volume) were filled with 200 μL of ^177^Lu solution and counted for 20 min in the well counter. The measurement duration was chosen in order to reach an acceptable statistical error (<1%) with a total number of counts higher than 10,000 in the photopeak for samples with the lowest activity. Background was measured daily and subtracted from the measurement [11]. No dead-time correction was applied.

**Fig. 3:**
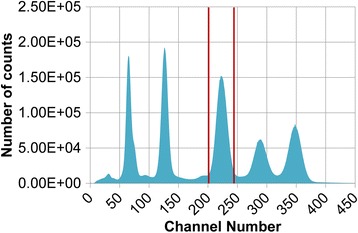
^177^Lu raw energy spectrum acquired on NaI(Tl) well counter. One energy window was defined around 208 keV photopeak (20% width)

The quantification was made by summing the number of counts detected in the 208 keV photopeak of the ^177^Lu with an energy window width of 20% of the energy of the peak as recommended by Zanzonico [12]. Raw data from the well counter (Fig. 3) were directly used to avoid spectrum modifications from the software part (peak position adjustment, energy spectrum resampling).

#### Calibration with ^177^Lu

The determination of a CF for the gamma counter was investigated. Standard tubes were filled with 200 μL of different amounts of ^177^Lu activities: six tubes were prepared with 130 kBq, two with 30 kBq, and three with 3 kBq. They were obtained with successive dilutions from a vial containing a known activity concentration of ^177^Lu: the volume was determined by measuring the weight of the vial before and after filling, and the activity was measured in the radionuclide calibrator. The 11 samples were measured eight times during 1.5 months, covering activities ranging from 130 to 0.15 kBq.

For each measurement, the CF expressed in cps/kBq was then computed as follows:

$$ \mathrm{C}\mathrm{F}=\frac{c_{\mathrm{mes}}}{T_{\mathrm{acq}}\times {A}_{\mathrm{prep}}\times \exp \left(\frac{- \ln 2\times \varDelta t}{T_{1/2}}\right)\ } $$

With *c*_mes_ the measured number of counts in the photopeak window, *T*_acq_ the acquisition duration, *A*_prep_ the prepared activity, *Δt* the time between the sample preparation and the acquisition, and *T*_1/2_ the physical half-life of the radionuclide. The final CF was obtained by computing the weighted mean value for the three groups of computed CFs ((6 + 2 + 3) samples × 8 measurements).

#### Quality control

The well counter sensitivity and the position of a reference energy peak were controlled monthly [11] with standard calibrated sources of ^129^I and ^137^Cs. Their main energy peaks (i.e., 40 and 662 keV, respectively) allowed checking the stability of the well counter over time for a large range of energies.

Specific quality controls were developed to evaluate the stability of the CF for ^177^Lu measurements over preparations and range of activities. For each patient treatment, the ^177^Lu-DOTATATE synthesis resulted in a vial containing 20 mL of radiopeptide. The activity in the vial was measured in the radionuclide calibrator, and the amount of activity needed for the patient treatment was adjusted by extracting a known volume from the vial with a syringe. For 34 treatments, a 200 μL sample with approximately 100 kBq was taken from the syringe containing a known activity concentration. The error was evaluated by comparing the well counter measured activity to the expected activity based on the radionuclide calibrator measurement corrected for physical decay.

Finally, among the 34 prepared samples, seven were measured five times during 2 months. Based on the established CF, their activities were computed over time. These results were corrected for physical decay and normalized by the mean value for each sample. It allowed evaluating the stability of the well counter over activities ranging from 120 to 0.2 kBq.

### Statistical analysis

Descriptive statistics are presented as $\mathsf{median}\left[\mathsf{interquartile}\ \mathsf{range}\right]$. On the one hand, the median value conveys the systematic error committed on activity quantification and the accuracy of the measurement compared to a reference. On the other hand, the interquartile range characterizes the random errors committed on activity quantification and the precision of the measurement. When box-and-whisker plots are used, the points outside the whiskers are mild outliers defined as values beyond *Q*1 − 1.5 × *IQ* and *Q*3 + 1.5 × *IQ* with *Q*1 and *Q*3 the first and third quartile of the considered distribution and *IQ* = *Q*3 − *Q*1 the interquartile range. Median and interquartile range are computed on all available data: the size of the considered population or sample is always referred to as *N*.

## Results

### Radionuclide calibrator

Based on the first ITG vial measurement, the CF was set to 150 × 10 for the CRC-15R radionuclide calibrator. 150 was the radionuclide calibrator dial setting for the ^177^Lu, and the readout had to be multiplied by a factor of 10 to obtain the activity value [13].

This factor was never modified. According to the specific implemented quality control, 93 vials were measured in total over 2.5 years. The systematic and random differences between the activity measured by the manufacturer and the on-site measurement were −0.99 [1.31] % (*N* = 93).

### SPECT/CT camera

**Fig. 4:**
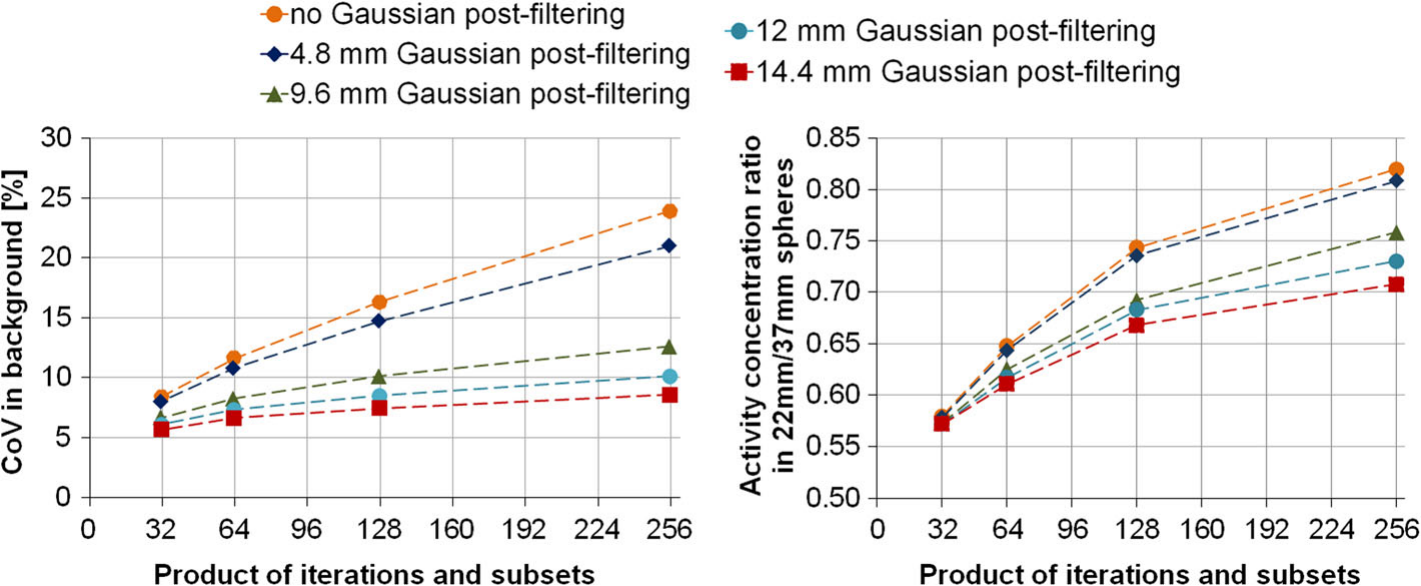
(*left*) The CoV in the main compartment of the NEMA phantom and (*right*) the activity concentration ratio between the 22 and the 37-mm spheres of the NEMA phantom on ^177^Lu SPECT/CT images with 0, 4.8, 9.6, 12, and, 14.4-mm filters and with 4i × 8s, 8i × 8s, 8i × 16s, and 16i × 16s

The CoV in the main compartment of the NEMA phantom and the partial volume effect on the spheres for different reconstruction parameters are presented in Fig. 4. The activity quantification ratio between the 22 and the 37 mm spheres was improved with the increasing number of iterations and subsets and decreasing filter size. The noise increased more rapidly with the increasing number of iterations and subsets for 0 and 4.8 mm filters. The best compromise corresponded to the maximum number of iterations and subsets (16i × 16s) with a 9.6-mm filter. Ultimately, after visual assessment of the reconstructed phantom images by the blinded expert nuclear medicine physician, the optimal parameters selected were the maximum number of iterations and subsets (16i × 16s) but with a 12-mm filter. Consequently, the later was used in future image reconstruction.

**Fig. 5:**
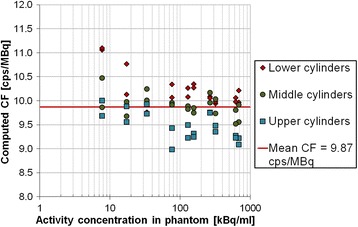
Computed SPECT CF for ^177^Lu activity concentrations and activities ranging from 686 to 8 kBq/ml and from 7040 to 80 MBq, respectively, (10 acquisitions) with 6 cylindrical VOIs equally distributed in lower, middle, and upper part of the NEMA phantom; the mean computed CF (*red line*)

The use of six VOIs in the main compartment of the NEMA phantom highlighted the importance of VOI location: the mean number of counts detected in the upper part of the phantom was systematically lower than in the bottom (Fig. 5). The final CF was set to 1.09 (cps/voxel)/(MBq/mL) by computing the mean value of the 60 calculated CFs (i.e., 6 VOIs × 10 acquisitions). For ease of comparison, this factor can also be expressed as 9.87 cps/MBq (*N* = 60), which is independent of the pixel size.

**Fig. 6:**
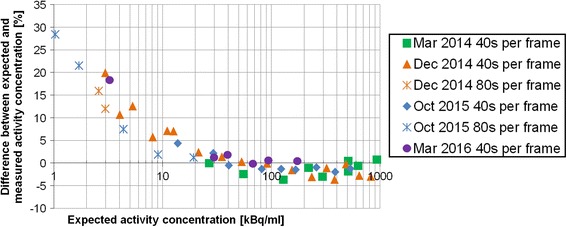
Differences, over time and range of activities, between expected and measured activity concentration in ^177^Lu uniform phantom area on SPECT/CT images. Activity concentrations and activities ranged from 933 to 1 kBq/ml and from 1926 to 0.6 MBq, respectively

For activity concentrations and activities ranging from 933 to 20 kBq/mL and from 1926 to 13 MBq respectively, the systematic and random errors committed on activity quantification were evaluated to −1.05 [2.12] % (*N* = 33) over 2.5 years when using the established CF (Fig. 6). For activity concentrations and activities lower than 20 kBq/mL and 13 MBq, respectively, the measurements highlighted an activity overestimation up to 30% (Fig. 6). Finally, one phantom acquired before and after the photomultipliers tuning on March 2014 showed a difference of 2.3% in the CF error.

### NaI(Tl) well counter

The CF for the well counter was set to 31.5 cps/kBq by computing the weighted mean value of 88 calculated CFs (i.e., (6 + 2 + 3) samples × 8 measurements). The standard deviation on all measurements was 1.14, yielding a CoV of 3.6%.

Based on the recommended measurements of ^137^Cs and ^129^I standard calibrated sources, the variability of the well counter sensitivity was evaluated to be less than 1% over 4 years.

Despite the high constancy of the sensitivity of the well counter over time, the systematic and random errors committed on activity quantification were estimated at −12.89 [16.55] % (*N* = 34) for measurements based on the residual patient activity.

**Fig. 7:**
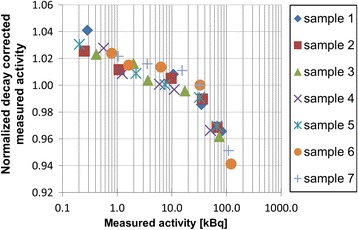
Decay-corrected measured activities in well counter normalized by the mean value for each sample over range of activities

A systematic underestimation (superior to 5%) was observed for activities higher than 100 kBq due to dead-time effect on quantification (Fig. 7).

## Discussion

Patient dosimetry offers the possibility of PRRT individualization with the prospect of improved therapy outcome. Owing that accuracy and precision of the activity measurements are prerequisites to a reliable dosimetry, we have studied the means to obtain them through the radionuclide calibrator, the SPECT/CT camera and the NaI(Tl) well counter. Quality requirements have been confronted to clinical setting constraints to define the optimal parameters for activity measurement for ^177^Lu-DOTATATE PRRT dosimetry. Home-made calibration sources and phantoms have been prepared using ^177^Lu, to compute the CF needed to convert measured counts to becquerels for each device. Finally, dedicated quality control steps have been proposed to evaluate the accuracy and the precision of the established CF for the chosen measurement parameters with respect to manufacturer maintenance, time, and range of activities. An error of −0.99 [1.31] % was found for the radionuclide calibrator activity quantification, demonstrating its high accuracy and precision over time. Therefore, it was considered as the reference for the preparation of the SPECT/CT camera phantoms and the well counter sources. The CF was established and controlled using the 2 mL manufacturer vial containing ^177^LuCl_3_, however, no control was performed on the 20 mL vial used in clinical routine for the ^177^Lu-DOTATATE production. Nonetheless, both vials were located in the same area of the radionuclide calibrator leading to comparable sensitivity. Besides, Beauregard et al. [8] have shown a reading difference of less than 0.2% between ^177^Lu solutions in syringes and Eppendorf tubes, stating that there was no significant geometry dependence. Although, the linearity of the CF was not systematically controlled over an array of ^177^Lu activities, it still was evaluated by means of a ^99m^Tc source measured from 30,000 to 0.5 MBq. The error committed on the computed half-life was 0.33%, and the maximum error on activity quantification was only 5% for the lowest measured activity (data not shown). This strengthened our confidence in the activity quantification reliability of the radionuclide calibrator over a range of activities used in clinical care.

The acquisition and reconstruction parameters for ^177^Lu SPECT imaging were studied and established in 2011, based on the paper of Beauregard et al. [8] and on the phantom measurements presented here. In 2012, the MIRD pamphlet No. 23, related to the standardization of SPECT imaging for molecular radiotherapy dosimetry, was published [14]. Although our study preceded this publication, most of our choices were in accordance to: body contouring, MELP collimator, 128 × 128 matrix with 4.8 mm pixel size, and total acquisition time of 25 min. The images were acquired in 64 frames of 40 or 80 s with a total acquisition time longer than reported by most patients’ studies [15–17]. On the other hand, to minimize undersampling, the number of frames should have been doubled to be equal to the matrix size [14]. Our SPECT acquisition approach was by way of the exclusive use of the 208 keV energy window. Other groups added the 113 keV photopeak, however, despite higher counting statistics, they showed that the contamination of the photopeak by scattered and down-scattered photons complicates the SC, occasioning increased noise [6, 7]. The OSEM reconstruction algorithm implemented with 16 iterations and 16 subsets, and a 12-mm FWHM Gaussian filter was also in accordance with the aforementioned MIRD pamphlet. These parameters offered a good compromise between noise and resolution and were suitable for clinical review as they were validated by an expert nuclear medicine physician based on visual image assessment. For lesion dosimetry, resolution is more critical and reconstruction parameters should probably be reassessed. Finally, the SC was implemented with the DEW method with *k* = 1 and 20 and 10% width for photopeak and scatter windows, respectively. It was supported by De Nijs et al. [10] who have implemented a similar DEW SC method with the same photopeak window width but with a scatter window twice as large and half the previous scaling factor (*k* = 0.5). For medium energy collimator, they have shown that the results were quite similar to those obtained with the triple energy window method and slightly weaker than with a more sophisticated effective scatter source estimation (ESSE) method [18]. Moreover, in the specific case of the ^177^Lu spectrum, the number of counts detected over 228.8 keV is not significant, avoiding the implementation of a triple energy window SC method based on the use of a third upper scatter window.

The SPECT CF was validated through repeated phantom measurements for activities and activity concentrations ranging from 7040 to 0.6 MBq and from 933 to 1 kBq/mL, respectively, over 2.5 years. The mean activity quantification error was −1.05 [2.12] % for activity concentrations higher than 20 kBq/ml. Consequently, dead time is not an issue for most centres where 7400 MBq fixed doses are commonly administered. However, PRRT personalization may require higher injected activities and dead-time problem will have to be readdressed. On the other hand, the measurement of activity concentrations lower than 20 kBq/ml highlights a systematic activity overestimation varying from a few percent up to 30% for an activity concentration of 1 kBq/mL. This can be a major concern for clinical applications where quantification is needed in areas of low activity concentration, such as the red marrow. The origin of the problem remains unclear but initial investigation suggests that a measured background signal could be subtracted to correct for this overestimation. The impact of photomultiplier tuning (during manufacturer maintenance) on activity quantification was evaluated as well and deemed not significant. Our study prompts the SPECT/CT camera as a highly reliable tool for activity quantification in PRRT. Furthermore, it stresses the importance of regularly controlling the constancy of the CF over time and its validity for activity concentrations encountered in patients’ studies.

Apart from the radionuclide calibrator and the SPECT/CT camera, the NaI(Tl) well counter is widely used in clinical setting in particular to quantify the activity in blood samples for bone marrow dosimetry in molecular radiotherapy [19, 20]. Unfortunately, measurement settings are rarely detailed and few recommendations are found. Moreover, the validation and practical implementation of relative or absolute calibration and quality controls are generally not reported. In this study, we have shown that the well counter was very constant, with less than 1% of variability on the relative number of counts detected over 4 years. It was measured with ^129^I and ^137^Cs standard calibrated sources as recommended [11].

The variability of the sensitivity over a range of activities was also evaluated and proved to be lower than 5% from 0.1 to 100 kBq of ^177^Lu. Over 100 kBq, count loss due to dead time becomes significant (Fig. 7). The activity in 200 μL patient blood samples is typically around 60 kBq just after the injection of ^177^Lu-DOTATATE. Thus, they are not impacted by this effect. This well-known dead-time problem and the well counter high sensitivity have been reported previously by Lodge et al. [21] and were also mentioned in the user manual. Consequently, for accurate quantification, samples need to contain an activity lower than 100 kBq. Such activity cannot be directly measured with any other instrument and has to be prepared by successive dilutions of a source measured in the radionuclide calibrator, adding in the process possible uncertainties on the sample activity.

The CoV of 3.6% on samples prepared for the computation of the CF gave high confidence in the reliability of the CF. However, important systematic and stochastic errors of −12.89 [16.55] % were measured for the samples issued from residual patient activity and prepared to control the CF. After investigation, it was concluded that the only significant difference between both cases was the use of ^177^Lu for the CF determination and of ^177^Lu-DOTATATE for the CF control. This hypothesis was further supported by the observation reported here. At the end of the ^177^Lu-DOTATATE preparation process, a certain volume of solution was extracted from the production vial so that the activity left in the vial corresponded to the intended activity to be injected to the patient. It was regularly observed that this extracted volume was larger than expected based on the known activity concentration in the production vial. It can only be the case if ^177^Lu-DOTATATE was not homogeneously distributed in the vial, even though the solution was vortexed during the process. The activity in samples taken from the syringe was too small to be measured by the radionuclide calibrator. Their activity was thus computed from the known activity concentration of the syringe, on the assumption that ^177^Lu-DOTATATE was homogeneously distributed. This assumption was likely false, leading to important errors in the activity determination of those samples. This highlights the importance of controlling every step of the sample preparation process for the well counter.

**Fig. 8:**
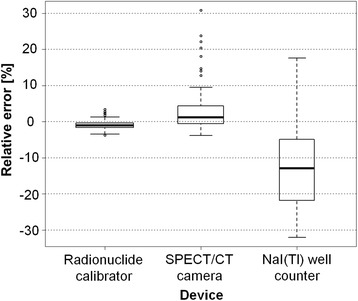
Box-and-whisker plot of the differences between the expected and the measured ^177^Lu activity for the radionuclide calibrator, the SPECT/CT camera and the NaI(Tl) well counter

Ultimately, the comparison of the relative quantification errors obtained with the radionuclide calibrator, the SPECT/CT camera and the NaI(Tl) well counter (Fig. 8) further supports the confidence in the quantification capabilities of ^177^Lu by the radionuclide calibrator and the SPECT/CT camera. Moreover, by working in the same range of activities, it allows easy cross-calibration with a unique radioactive source. Due to its very high sensitivity, the well counter can only be calibrated after dilution or decay of the aforementioned source or with dedicated calibrated source. Despite this weakness, the well counter is a very precise measurement tool making wide range of activities handling possible.

## Conclusions

This study proposed critical and dedicated quality control steps related to the quantification of the ^177^Lu activity with a radionuclide calibrator, a SPECT/CT camera and a NaI(Tl) well counter. The appropriate use of these measuring devices has been validated in a clinical setting and results in precise and accurate activity quantification. Based on these activity measurements, reliable absorbed dose computation can be developed for ^177^Lu-based therapy. More importantly, our findings have highlighted the importance of the knowledge of the strengths and weaknesses of the measuring devices involved, as well as the errors committed on activity quantification. This information is indeed of utmost importance to obtain reliable results and should therefore always be investigated in further developments of dosimetry protocols.
